# Improving catheter navigation in neurointerventional procedures: single-center insights on next-generation steerable guidewires

**DOI:** 10.1007/s00234-026-03907-y

**Published:** 2026-01-24

**Authors:** Om H. Gandhi, Suraj R. Dumasia, Sami Almasri, Nathan Yu, Erin N. Walker, Linda Bagley, Omar A. Choudhri

**Affiliations:** 1 Department of Neurosurgery, University of Pennsylvania, Philadelphia, PA USA; 2 Department of Radiology, University of Pennsylvania, Philadelphia, PA USA

**Keywords:** Steerable guidewire, Neurointerventional, Cerebrovascular navigation, Aneurysm treatment, Stroke intervention

## Abstract

**Purpose:**

Steerable guidewires with deflectable tips enable real-time navigation through complex cerebrovascular anatomy without requiring wire removal and tip reshaping. Despite promising early reports, systematic clinical evaluation remains limited.

**Methods:**

We conducted a retrospective analysis of 58 consecutive neurointerventional procedures utilizing steerable guidewires at two centers from September 2024 to August 2025. The cohort comprised 48 cases using the Drivewire 24 (DW24, 0.024-inch diameter) and 10 cases using the Artiria SmartGUIDE (0.014-inch diameter). Primary outcomes included technical success, safety profile, and operator assessment. We also performed a scoping literature review of all published steerable guidewire studies in neurointerventional applications.

**Results:**

Technical success in reaching target vessels was achieved in 100% of cases for both systems with no intraoperative or postoperative complications. Mean fluoroscopy time was 32.7 ± 29.5 min for DW24 and 26.1 ± 16.2 min for Artiria procedures. Treatment indications included aneurysm procedures (53.4%), diagnostic angiographies (20.7%), venous sinus stenting (13.8%), middle meningeal artery embolizations (8.6%), and stroke interventions (3.4%). 8.3% of procedures using DW24 were considered higher-risk by the primary operator without steerable capability, including successful navigation of complex posterior circulation anatomy in giant basilar aneurysms and recurrent superior cerebellar artery aneurysms with prior stent constructs. Our limited experience with the Artiria system (*n* = 10) revealed operator-noted challenges with shape retention. The scoping review identified only three prior studies encompassing 65 procedures, establishing this as the largest reported experience.

**Conclusion:**

Both steerable guidewire systems achieved perfect technical success with excellent safety profiles. Based on our preliminary experience, the DW24 appeared to offer advantages for procedures requiring navigating larger catheters through tortuous anatomy. These findings support selective clinical use of steerable guidewire technology in challenging neurointerventional procedures.

**Supplementary Information:**

The online version contains supplementary material available at 10.1007/s00234-026-03907-y.

## Introduction

 Neurointerventional procedures rely on precise guidewire navigation through complex cerebrovascular anatomy to deliver therapeutic devices such as stents, coils, flow diverters, and aspiration catheters [1]. Traditional guidewires are limited by their fixed tip configurations that cannot be modified in real-time during procedures. When navigating tortuous anatomy, optimal positioning often requires tip reshaping where clinicians must remove the guidewire from the patient, manually reshape the tip extracorporeally, and reinsert the device. This repositioning leads to loss of established vascular access, increased procedure time/radiation exposure, and potential complications from repeated navigation attempts [2]. Although robotic-assisted systems have been developed that provide remote advancement and torquing capabilities from a radiation-shielded cockpit, these platforms still rely on conventional fixed-tip guidewires and cannot alter tip geometry when encountering complex anatomy [3]. In contrast, steerable guidewires address these limitations through a fundamentally different approach by incorporating deflectable tips that can be actively shaped and manipulated in real-time while maintaining intravascular position.

Steerable guidewire technology initially gained traction in interventional cardiology, where similar navigation challenges in coronary vasculature drove the development of three distinct steering approaches: standard electromagnetic navigation, magnetic microrobots, and handle-controlled microcatheters/guidewires. The Stereotaxis Niobe magnetic navigation system pioneered remote guidewire control using two permanent magnets positioned on opposite sides of the patient table, creating a controllable magnetic field that enables vector-based navigation of magnetically-enabled guidewires, which achieved successful lesion crossing in 77–100% of cases across multiple case studies [4–10]. However, the device faced challenges regarding chronic total occlusions (CTO) with 71% (25/35) of failures attributed to CTOs in the largest series [5], similar to navigating chronically occluded intracranial vessels or calcified stenotic lesions that present comparable resistance to guidewire advancement in cerebrovascular interventions. Newer magnetic approaches include magnetically actuated soft microrobots—millimeter-scale devices fabricated from polydimethylsiloxane with embedded permanent magnets—attach to standard 0.014-inch guidewire tips and deform in response to external electromagnetic fields, achieving steering angles from 21° to 133° at field intensities as low as 15 mT [11–18]. These newer magnetic technologies proved particularly valuable in scenarios where conventional guidewires struggle such as CTOs with > 45° angulation, vessels with multiple tortuous segments, and side branch access through stent struts. In recent years, steerable guidewires and microcatheters have emerged as an alternative solution, with devices like the STORQ™ Steerable Guidewire (Cordis), Venture Wire Control, and SwiftNINJA (MERIT medical) incorporating mechanically activated deflectable tips that bend 90–180° via handle manipulation, enabling navigation through extreme coronary angulations and vessel tortuosity where conventional guidewires alone cannot reach [19–28]. The success of these platforms in coronary intervention provided the technological foundation for adaptation to cerebrovascular applications.

Several next-generation guidewires, steerable via manual handle control, have since been developed specifically for neurointerventional use. The Columbus steerable guidewire (Rapid Medical, Israel), a 0.014-inch diameter device, was among the earliest innovations featuring a remotely controlled deflectable tip [29, 30]. While it demonstrated the feasibility of steerable technology in neurointervention, clinical use revealed significant limitations including fragile construction and poor response to rotational force, leading to its discontinuation. The Drivewire 24 (DW24), also from Rapid Medical, advances the field with its more robust 0.024-inch diameter and innovative deflectable tip mechanism that allows operators to curve or straighten the tip via handle manipulation without compromising established vascular access [31]. Most recently, Artiria’s SmartGUIDE, a 0.014-inch diameter device, received FDA clearance, featuring real-time steering capabilities through an external control unit that allows continuous adjustment of guidewire tip orientation during navigation [32, 33]. 

Early clinical reports suggest these steerable guidewires can successfully access challenging cerebrovascular anatomy where conventional guidewires fail, such as reaching distal M2 occlusions in acute stroke interventions [30]. Success rates as high as 92.6% have been reported for the DW24; however, despite these encouraging technological features and initial reports, systematic evaluation of steerable guidewires in neurointerventional practice remains limited [31]. The current literature consists primarily of single-center case series and technical reports focused on the DW24 and earlier Columbus devices, with validation across broader clinical experiences still needed. Furthermore, no peer-reviewed clinical studies exist evaluating the Artiria SmartGUIDE system, and limited data exists comparing the performance characteristics and clinical applications of different steerable guidewire platforms in current use.

This study presents the largest single-center experience with next-generation steerable guidewires in neurointerventional procedures, evaluating the safety, efficacy, and ease of use of both the DW24 and SmartGUIDE systems in clinical practice. Additionally, we present a scoping literature review of all published steerable guidewire studies in neurointerventional applications to contextualize our institutional experience within the broader body of evidence. Through analysis of procedural outcomes, technical performance, and operator feedback, this work aims to provide practical insights for clinicians considering the integration of these advanced navigation tools into their neurointerventional practice.

## Methods

### Guidewire & technique details

**Fig. 1:**
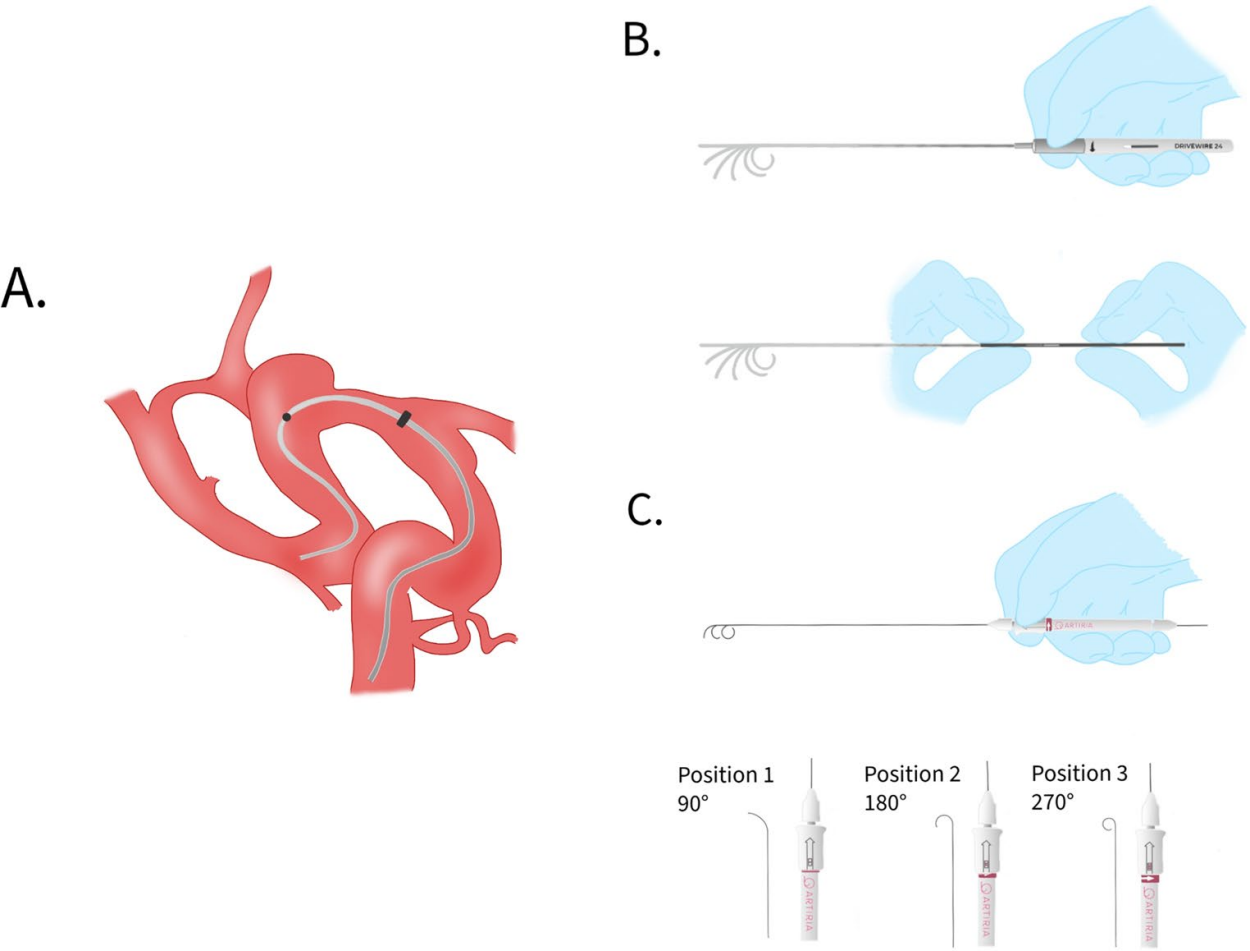
Steerable guidewire technology and tip deflection mechanisms in cerebrovascular navigation. (**A**) Schematic demonstration of steerable guidewire navigation through tortuous cerebrovascular anatomy, illustrated with a fusiform right middle cerebral artery aneurysm. The deflectable tip enables real-time navigation without requiring wire removal and extracorporeal reshaping. (**B**) Drivewire 24 (DW24, Rapid Medical, Yokneam, Israel) tip deflection mechanisms showing two operational approaches: single-hand technique using a custom torque handle with graded spectrum of wire-tip curves achieved by rotating the top portion counterclockwise (arrow), and two-hand technique manipulating curve by adjusting proximal handle contact distance. The 0.024-inch diameter device features a 15.7 mm deflectable tip segment that can be shaped into a loop with a 5 mm bend diameter when fully deflected. (**C**) Artiria SmartGUIDE (Artiria Medical, Borex, Switzerland) deployment technique using specialized torque device with three fixed tip curve positions: 90° (single line in deployment window), 180° (two lines), and 270° (three lines), adjusted by sliding and rotating the top portion clockwise. The 0.014-inch diameter device incorporates a patented micromechanical system enabling real-time tip deflection control

Steerable guidewires utilize controlled tip deflection mechanisms to navigate complex cerebrovascular bifurcations and tortuous vessel segments (Fig. 1A). Two next-generation steerable guidewire systems were evaluated in this study: the Drivewire 24 (DW24, Rapid Medical Ltd, Yokneam, Israel) and the Artiria SmartGUIDE (Artiria Medical, Borex, Switzerland). Complete technical specifications for both devices are provided in Table S1, which also includes specifications for the Columbus steerable guidewire (Rapid Medical Ltd) that is no longer in clinical use.

The DW24 is a 0.024-inch diameter steerable guidewire with a total length of 204 cm. The device features a 15.7 mm deflectable tip that can be shaped into a loop with a 5 mm bend diameter when fully deflected. The proximally controlled deflectable tip is manipulated via a handle mechanism that allows for variable radius control with automatic position maintenance (Fig. 1B). The tip exhibits softer characteristics similar to those of conventional Synchro Support wires, while the shaft incorporates a variable support profile with an internal support mechanism. The distal 40 cm of the wire is radiopaque for enhanced visualization, with a fluorosafe marker positioned 100 cm from the distal end. A hydrophilic coating on the distal tip facilitates smooth navigation through tortuous anatomy. The DW24 is designed for compatibility with 0.024-inch microcatheters and can be delivered without requiring a wire forerun. The device received FDA clearance in 2024 and CE marking in September 2025. It is the only FDA-approved 0.024-inch diameter steerable wire currently available.

During the study period, the DW24 system underwent a design evolution in its deflection control mechanism. The initial 38 cases used an earlier iteration that required two-handed manipulation to bring junction points together for tip deflection. The final 10 cases used an updated handle-controlled system that allowed single-handed operation for tip steering. Both versions maintained the same core technical specifications including the 0.024-inch diameter, 15.7 mm deflectable tip length, and 5 mm bend diameter capabilities (Fig. 1B).

The Artiria SmartGUIDE is a 0.014-inch diameter steerable guidewire with a total length of 200 cm. The device incorporates a patented micromechanical system that enables real-time tip deflection control through a manipulator handle system, allowing precise positioning at three distinct angles (90°, 180°, and 270°) (Fig. 1C). Unlike fixed-curvature steerable wires, the SmartGUIDE allows dynamic, real-time adjustment of tip orientation during navigation between these three predetermined positions. The soft atraumatic tip is designed to provide enhanced support when deflected while maintaining compatibility with 0.014-inch microcatheters. The system eliminates the need for wire forerun during catheter delivery and claims to provide 3× faster navigation in cerebral arteries compared to conventional guidewires. The device received FDA 510(k) clearance in May 2023, with first-in-human trials completed in Q3 2023. CE mark evaluation is currently under review.

Steerable guidewire technique involved initial advancement of the wire in its straight configuration through the guide catheter to the target vessel origin, followed by activation of the tip deflection mechanism to achieve the desired curvature for vessel selection. For the DW24, tip deflection was controlled via the handle mechanism, allowing gradual adjustment from straight to fully curved configurations. For the Artiria SmartGUIDE, preset deflection angles (90°, 180°, or 270°) were selected using the external control unit. Once target vessel access was achieved, the deflectable tip was typically straightened to facilitate microcatheter advancement. Technical success was defined as successful navigation of the steerable guidewire to the target vessel with subsequent successful catheter advancement over the wire. All DW24 cases and 5 of the 10 Artiria cases were performed by a single dual-trained vascular neurosurgeon; the remaining 5 Artiria cases were performed by other operators. Operators noted a learning curve of approximately 3–5 cases to achieve proficiency with each device, particularly in coordinating tip deflection with wire torque during navigation of acute vessel angulations.

### Study cohort & data collection

We conducted a retrospective review of 58 consecutive neurointerventional cases utilizing steerable guidewires from September 2024 to August 2025. The cohort comprised 48 cases using the DW24 and 10 cases using the Artiria SmartGUIDE wire. The majority of cases were performed by a single dual-trained vascular neurosurgeon, who performed all DW24 cases and 5 of the Artiria cases.

Data collection included patient demographics (age, sex, race), medical comorbidities (hypertension, hyperlipidemia), smoking status, treatment indications, vascular access approach, and catheter types utilized. Wire usage patterns were documented, including primary wire designation and concurrent use of additional wires. Technical performance metrics included fluoroscopy time, technical success rates, safety outcomes (intraoperative and postoperative complications), and antiplatelet therapy administration. Operator assessment included usability ratings, procedures deemed impossible without steerable wire capability, and comparative performance characteristics between wire platforms. All data were collected from electronic medical records and procedural documentation.

### Scoping literature review

Studies discussing steerable guidewires in neurointerventional procedures were reviewed by searching PubMed and Google Scholar using terms including “steerable guidewire,” “Columbus guidewire,” “Drivewire,” “Artiria SmartGUIDE,” and “neurointerventional procedures.” Only peer-reviewed articles reporting clinical experience with steerable guidewires in human subjects undergoing neurointerventional procedures were included. Only three studies were identified: two retrospective case series and one technical video demonstration, encompassing 65 total procedures across 64 patients treated between August 2019 and April 2025. No peer-reviewed clinical studies on the Artiria SmartGUIDE were found. Data on device specifications, technical success rates, procedural indications, complications, operator feedback, and clinical outcomes were extracted from eligible studies.

## Results

This study analyzed 58 consecutive neurointerventional procedures utilizing steerable guidewires, comprising 48 cases with the DW24 system and 10 cases with the Artiria SmartGUIDE wire. The DW24 cohort included 30 female patients (62.5%) with a mean age of 61.1 ± 13.1 years (range 32–83 years). Racial distribution was predominantly White (58.3%), followed by Other/Unknown (25%), Black (12.5%), and Asian/Pacific Islander (4.2%). Common comorbidities included hypertension in 31 patients (64.6%) and hyperlipidemia in 31 patients (64.6%). Smoking history revealed that 22 patients (45.8%) were never smokers, 20 (41.7%) were former smokers, and 6 (12.5%) were current smokers. The Artiria cohort included 9 female patients (90%) with a mean age of 58.9 ± 15.3 years (range 37–78 years). This cohort showed equal representation of White and Black patients (40% each), with 10% Asian/Pacific Islander and 10% Other/Unknown. Hypertension was present in 6 patients (60%) and hyperlipidemia in 6 patients (60%). Smoking history included 2 never smokers (20%), 6 former smokers (60%), and 2 current smokers (20%) (Table 1).

**Table 1:** Summary of demographic and operative data

Variable	DriveWire 24 (*n* = 48)	Artiria Wire (*n* = 10)
**Demographics**		
Female	30 (62.5%)	9 (90%)
Male	18 (37.5%)	1 (10%)
**Race**		
White	28 (58.3%)	4 (40%)
Black	6 (12.5%)	4 (40%)
Asian/Pacific Islander	2 (4.2%)	1 (10%)
Other/Unknown	12 (25%)	1 (10%)
**Age at Surgery**		
Mean ± SD (years)	61.1 ± 13.1	58.9 ± 15.3
Range (years)	32–83	37–78
**Comorbidities**		
Hypertension	31 (64.6%)	6 (60%)
Hyperlipidemia	31 (64.6%)	6 (60%)
**Smoking Status**		
Never	22 (45.8%)	2 (20%)
Current	6 (12.5%)	2 (20%)
Former	20 (41.7%)	6 (60%)

Procedural characteristics demonstrated the versatility of steerable guidewire technology (Table 2). Both wire systems served as the primary wire in all cases (100%). The DW24 system was used with one additional wire in 28 cases (58.3%), two additional wires in 15 cases (31.3%), and three additional wires in 4 cases (8.3%). The Artiria system was used with one additional wire in 9 cases (90%) and two additional wires in 1 case (10%). Vascular access for DW24 cases was predominantly via the right radial artery in 47 cases (97.9%) and the right femoral artery in 1 case (2.1%). Artiria cases utilized right radial artery access in 6 cases (60%), right groin access in 3 cases (30%), and right cephalic vein access in 1 case (10%). Treatment indications for the DW24 system included aneurysm procedures in 22 cases (45.8%), diagnostic angiographies in 12 cases (25.0%), Middle Meningeal Artery (MMA) embolizations in 5 cases (10.4%), venous sinus stenting in 7 cases (14.6%), and stroke interventions in 2 cases (4.2%). Artiria cases comprised aneurysm procedures in 9 cases (90%) and venous sinus stenting in 1 case (10%). Catheter compatibility data were available for the DW24 system, showing successful navigation with XT-27 catheters (27.1%), Sim-2 Glide catheters (25%), 5 F Berenstein catheters (10.4%), 6 F Sofia catheters (8.3%), and various other catheter systems (29.2%).

**Table 2:** Summary of procedural characteristics and outcomes

Variable	DriveWire 24 (*n* = 48)	Artiria Wire (*n* = 10)
**Wire Usage**		
Primary wire	48 (100%)	10 (100%)
Used with 1 additional wire	28 (58.3%)	9 (90%)
Used with 2 additional wires	15 (31.3%)	1 (10%)
Used with 3 additional wires	4 (8.3%)	0 (0%)
**Vascular Access**		
Right radial artery	47 (97.9%)	6 (60%)
Right femoral artery	1 (2.1%)	3 (30%)
Right cephalic vein	0 (0%)	1 (10%)
**Treatment Indications**		
Aneurysms	22 (45.8%)	9 (90%)
Diagnostic angiographies	12 (25%)	0 (0%)
Venous sinus stenting	7 (14.6%)	1 (10%)
MMA embolizations	5 (10.4%)	0 (0%)
Stroke intervention	2 (4.2%)	0 (0%)
**Catheters Used**		
Sim-2 Glide	12 (25%)	-
XT-27	13 (27.1%)	-
5 F Berenstein	5 (10.4%)	-
6 F Sofia	4 (8.3%)	-
Others	14 (29.2%)	-
**Procedural Outcomes**		
Fluoroscopy Time (mean ± SD, min)	32.7 ± 29.5	26.1 ± 16.2
P2Y12 inhibitors/DAPT used	46 (95.8%)	10 (100%)
Technical success	48 (100%)	10 (100%)
Intraoperative complications	0 (0%)	0 (0%)
Postoperative complications	0 (0%)	0 (0%)
**Operator Assessment**		
Rated as easy to use	48 (100%)	10 (100%)
Procedures deemed impossible/high-risk without wire	4 (8.3%)	-

Both wire systems demonstrated excellent technical performance and safety profiles. Technical success in reaching target vessels was achieved in 100% of cases for both cohorts, with no intraoperative or postoperative complications reported. Mean fluoroscopy time was 32.7 ± 29.5 min for DW24 procedures and 26.1 ± 16.2 min for Artiria procedures. Antiplatelet therapy utilization was high, with P2Y12 inhibitors or dual antiplatelet therapy administered in 46 DW24 cases (95.8%) and all 10 Artiria cases (100%).

Operator assessment revealed important performance differences between the two steerable guidewire systems. While both wires were rated as easy to use in 100% of cases, the Artiria wire had limitations in shape maintenance, with restriction to only three shape variations, highly variable performance depending on extent of torque tightening, and less support when working with larger intermediate and microcatheters due to its inherently smaller diameter compared to the 0.024-inch DW24 system. Additionally, the primary operator subjectively assessed that 8.3% of procedures would have been considered higher-risk without the DW24 capability. Two cases involving complex posterior circulation aneurysms that were challenging without DW24 are described:

### Illustrative case 1

**Fig. 2:**
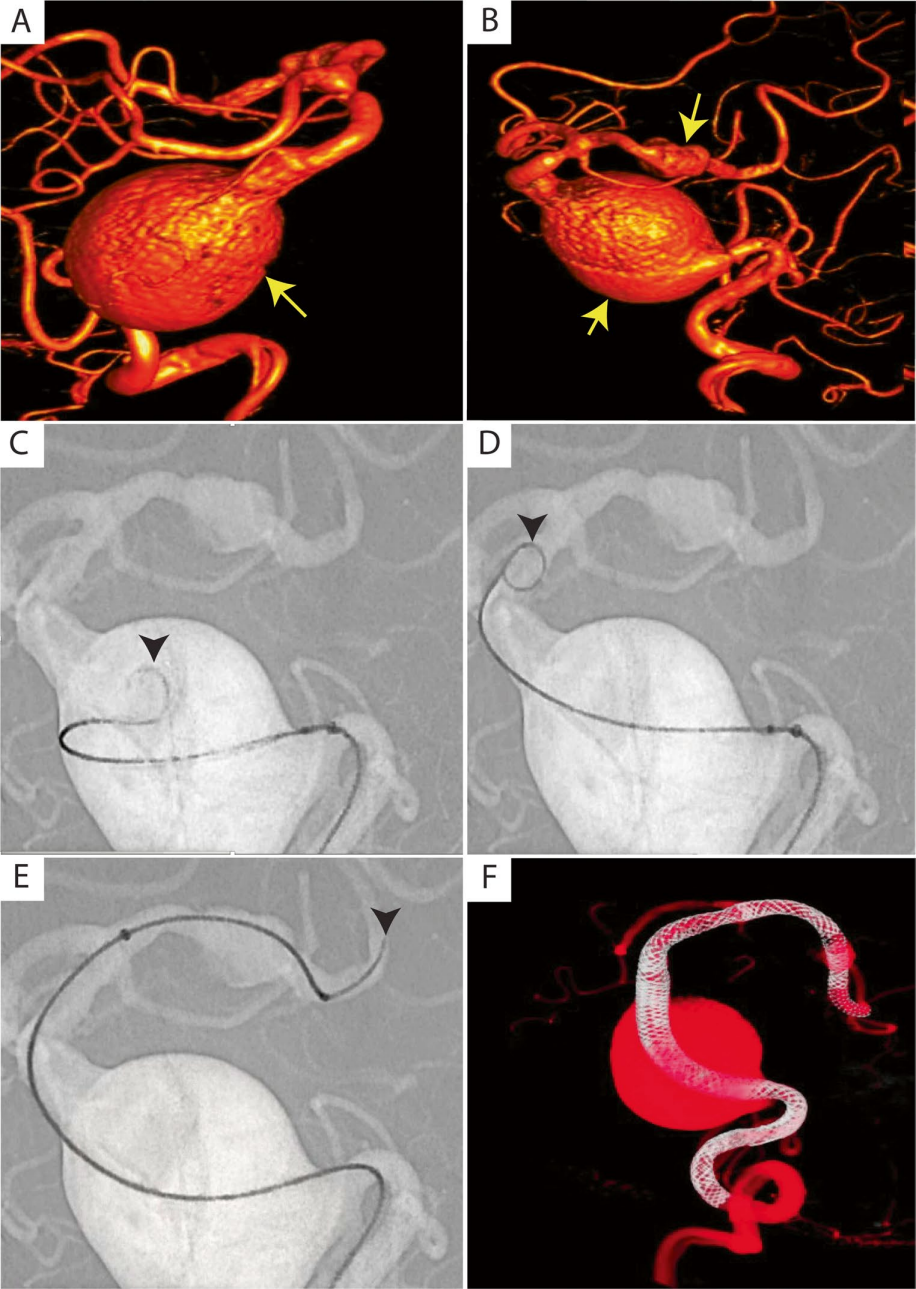
Complex posterior circulation navigation using DW24 with multiple tip configurations in challenging anatomy. Case demonstrating navigation of a dolichoectatic giant basilar trunk and fusiform left posterior cerebral artery (PCA) P2A segment aneurysm using DW24 (Rapid Medical, Yokneam, Israel). (**A** & **B**) Oblique rotational 3D angiogram reconstructions from left vertebral artery injections showing the dolichoectatic basilar aneurysm and left PCA aneurysm (yellow arrows). (**C**, **D**, **E**) DW24 navigation under roadmap working projection views across the aneurysm demonstrating three different tip configurations switched in-vivo at different navigation points (black arrowheads). (**F**) Post-treatment dual volume rotational 3D angiogram reconstruction showing robust long-segment flow diverter construct with multiple Surpass Elite flow diverters (Stryker Neurovascular) across diseased aneurysmal segments

A 67-year-old female with a history of hypertension and ischemic cardiomyopathy was found to have a giant dolichoectatic basilar artery aneurysm and a left PCA aneurysm during workup for syncope. The patient underwent aneurysm treatment of both aneurysms using a flow divertor construct via right radial artery access. The DW24 steerable guidewire successfully navigated through challenging anatomy, reaching the left P2 segment and basilar artery. Catheter navigation was accomplished using an XT-27 catheter and Vecta 46 system advanced over the DW24 wire. The DW24 allowed successful navigation across tortuous giant aneurysm anatomy to successfully locate the outflow zone of the aneurysm. A total of 11 Surpass Elite (Stryker) flow divertors were successfully deployed spanning the aneurysmal segments. Two additional wires were required during the procedure: a 0.014” Synchro 2 microwire to accompany the Eclipse dual balloon (Balt) for flow divertor post-processing, and a 0.035” Glidewire to navigate the guide catheter construct into the vertebral artery. Total fluoroscopy time was 43 min. The DW24 demonstrated easy usability with successful access to target vessels and no device-related perforations or intraoperative complications. It obviated the need for complicated high-risk maneuvers such as “going around the world” to find the aneurysm outflow. The supportive characteristics of the wire helped keep the microcatheter straight and allowed ease in advancing the Vecta 46 intermediate catheter over it. Post-procedural angiography showed no significant filling of the right vertebrobasilar junction with preserved right posterior inferior cerebellar artery (PICA) patency. The patient was maintained on dual antiplatelet therapy with aspirin and clopidogrel in addition to apixaban and experienced no postoperative complications (Fig. 2).

### Illustrative case 2

**Fig. 3:**
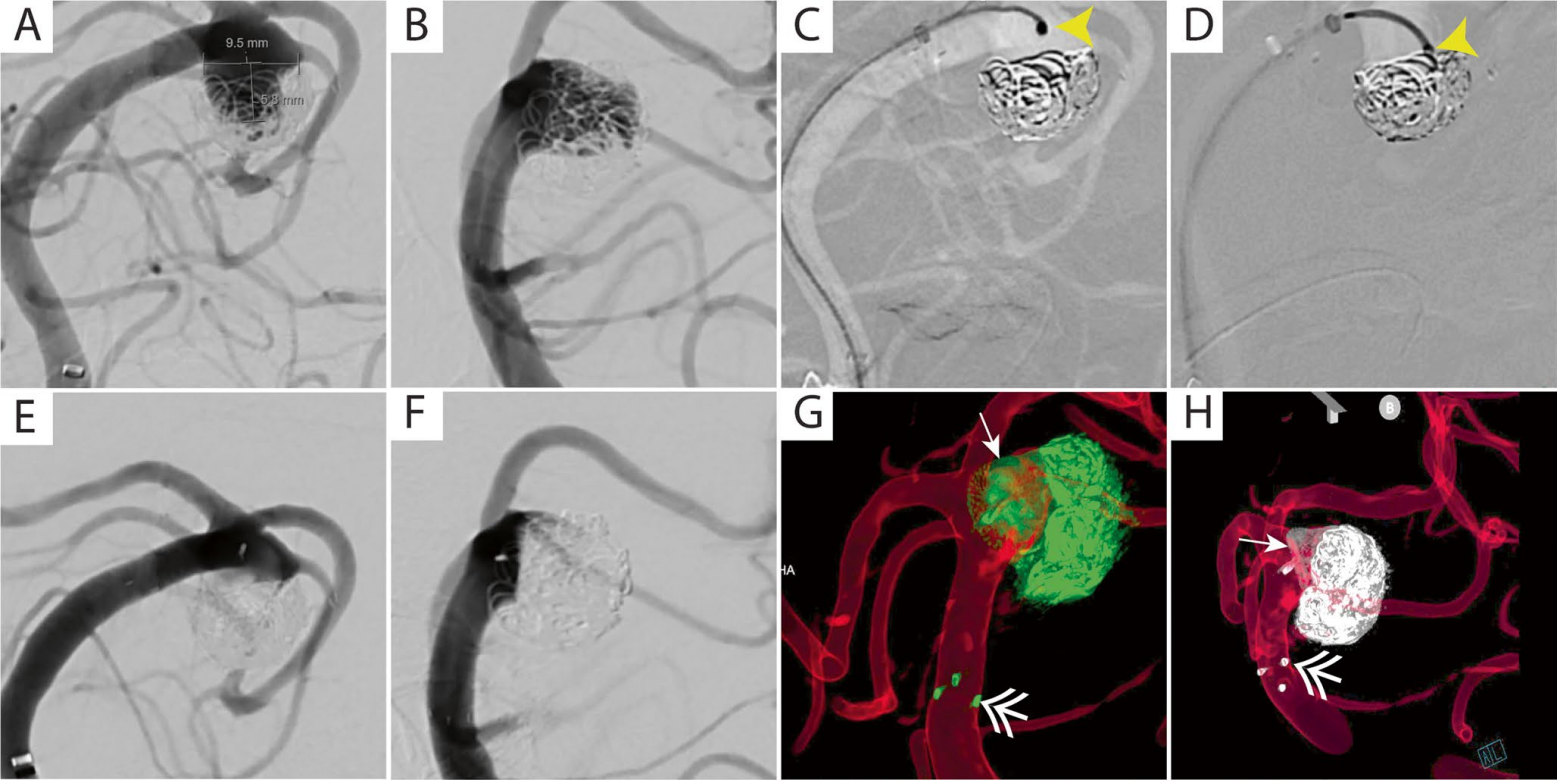
DW24-assisted deployment of large WEB intrasaccular device at acute angle through prior stent construct. Challenging case involving a 12x9mm aneurysm recurrence at the base of a previously stent-coiled left superior cerebellar artery (SCA) aneurysm with coil compaction. (**A** & **B**) AP and lateral oblique working projection angiographic views from vertebral artery injections showing aneurysm recurrence projected posteriorly and inferiorly at 90° to the parent basilar artery. (**C** & **D**) Deployment of 10 × 5 mm Woven EndoBridge (WEB) device required aneurysm selection with straight-tip VIA 33 microcatheter. Sharp curve navigation (yellow arrowheads) and added DW24 (Rapid Medical, Yokneam, Israel) support enabled navigation through prior Neuroform ATLAS stent (Stryker Neurovascular) struts for stable WEB deployment. (**E** & **F**) Immediate post-treatment AP and lateral oblique views showing excellent aneurysm obliteration with parent vessel preservation. (**G** & **H**) Post-treatment dual volume rotational 3D reconstructions demonstrating optimal WEB device placement (white arrows) and proximal markers of prior Neuroform ATLAS stent (white double arrows)

An 82-year-old White female with a medical history of hypertension, hyperlipidemia, Bell’s palsy, and a previously treated 9 mm left superior cerebellar artery aneurysm (status post stent-assisted coiling 3 years earlier) presented for treatment of aneurysm regrowth. The patient had developed coil compaction with enlargement of the left superior cerebellar artery (SCA) aneurysm, which had increased in size to 1.2 × 0.9 cm. Treatment was performed via right radial artery access using an intrasaccular WEB device. The DW24 steerable guidewire served as the primary wire and successfully navigated challenging anatomy to reach the left SCA segment. Catheter navigation was accomplished using a Via 33 microcatheter and 6 F Sofia system advanced over the DW24 wire. The Via 33 microcatheter is the largest neurointerventional microcatheter and is extremely difficult to shape given its caliber and construction. We attempted to steam-shape the microcatheter, but it failed to maintain its shape. The aneurysm was positioned eccentrically to the left, and microcatheterization with the Via 33 required a supportive wire and additional shape robustness to navigate through the interstices of the previously placed ATLAS stent. We were able to achieve this only with the DW24, which initially allowed a tight tip curve to pass through the prior stent interstices and enter the residual aneurysm, successfully enabling navigation of the Via 33 catheter over the wire and maintaining its position while a large intrasaccular device was deployed. A 10 × 5 mm Woven EndoBridge (WEB) device was successfully deployed using the DW24 system for aneurysm treatment. One additional wire was utilized during the procedure to navigate into the vertebral artery, with a total procedure fluoroscopy time of 49.8 min. Post-procedural angiography demonstrated ideal positioning of the intrasaccular device with early aneurysm obliteration and contrast stasis. The DW24 demonstrated easy usability with successful access to the target vessel and no device-related perforations or intraoperative complications. The patient was maintained on single antiplatelet therapy with aspirin and experienced no postoperative complications (Fig. 3).

## Discussion

To our knowledge, this study is the largest single-center experience with next-generation steerable guidewires in neurointerventional procedures and provides the first peer reviewed clinical data on the Artiria SmartGUIDE system. Our literature review identified only three peer-reviewed studies reporting clinical experience with steerable guidewires in neurointerventional procedures, encompassing 65 total procedures across 64 patients (Table S2). These cases consisted of 27 cases using DW24 system from Grin et al. and 36 cases utilizing the Columbus guidewire from von Hessling et al. along with 2 technical demonstrations [29–31]. All previous studies were single-center experiences with small sample sizes spanning August 2019 to April 2025. Previous indications focused primarily on aneurysm treatment and stroke thrombectomy, with technical success rates ranging from 92.6% to 100%. Device-related complications occurred exclusively with the Columbus system in 5 of 36 cases (13.9%), including handle detachment in 2 cases and wire damage in 5 cases during operator manipulation [30], while DW24 studies reported no device failures [31]. 

Our findings substantially expand upon this limited literature base in several important ways. We achieved perfect technical success rates with both wire systems, surpassing or matching previously reported rates despite employing steerable guidewires across a broader range of procedural indications. Beyond the aneurysm treatments and stroke interventions that dominated earlier studies, we successfully applied steerable guidewires to diagnostic angiographies, venous sinus stenting, and MMA embolizations. Additionally, our predominant use of radial access contrasts with the exclusive femoral access reported in previous series, likely reflecting the increased adoption of radial-first approaches in neurointerventional procedures and compatibility with newer steerable wire platforms. Our early experience with both systems suggested some differences in handling characteristics despite both achieving perfect technical success. The DW24’s larger diameter provided enhanced support when navigating larger intermediate and aspiration catheters, with the primary operator noting that a subset of procedures would have been considered higher-risk without this specific steerable capability. In contrast, the primary operator rated the Artiria SmartGUIDE as having less reliable shape retention, poorer reusability, and restriction to only three shape variations. These limitations of the Artiria system parallel the torquability issues reported with the earlier Columbus system, while the DW24 appears to have successfully addressed many of these concerns [29–31]. 

The clinical value of steerable guidewires becomes particularly evident when navigating acute angulations with heavy catheter systems in challenging posterior circulation anatomy. In our giant basilar aneurysm cases, the DW24’s deflectable tip was essential for traversing the geometric challenges associated with finding outflow from giant dolichoectatic basilar aneurysms. Case 1 required navigation from the vertebrobasilar junction through a large basilar-left PCA aneurysm to reach the left P2 segment, a path complicated by the aneurysm’s mass effect creating sharp directional changes at the P1 origin and the acute angulation typically encountered at the P1-P2 junction. The steerable tip allowed real-time adjustment to accommodate the nearly 90-degree turn from the basilar artery into the left P1 segment, then the subsequent sharp angulation into P2, while supporting an XT-27 microcatheter and Vecta 46 system for Surpass Elite deployment. Case 2 involved accessing the left superior cerebellar artery recurrent aneurysm with a prior Neuroform ATLAS stent in place. This target is particularly challenging due to its acute takeoff angle from the distal basilar artery, often requiring precise tip positioning within the narrow corridor between the aneurysm neck and the SCA ostium. The 0.024-inch diameter provided crucial support for the Via 33 microcatheter and 6 F Sofia system through these tight spaces, while the deflectable tip maintained stable purchase at the SCA origin during WEB device deployment. These anatomical scenarios—eccentric sidewall aneurysms with acute takeoff angles, prior stents in place, and geometric vessel outflow challenges—represent situations where conventional wires frequently fail due to inability to maintain both directional control and adequate support. The steerable capability also eliminated the typical need for removing the wire multiple times to introduce different tip shapes before reintroduction during different parts of the case. The DW24 with its inherent tip retention is particularly valuable given the tendency for conventional wires to prolapse back into the vertebral system when encountering the acute angles at these critical bifurcation points.

Understanding the broader context of steerable catheter technology in neurointerventional procedures provides important perspective on steerable guidewire development. Steerable catheters incorporate deflectable tips that can be manipulated in real-time through external controls, in contrast to steerable guidewires, which provide directional navigation within existing catheter systems rather than serving as the primary therapeutic delivery platform. Earlier steerable microcatheter systems like the Enzo (Micrus Endovascular Corporation) had significant limitations such as wide turn radius and poor torque transmission that restricted clinical utility [34, 35]. The Bendit steerable microcatheter system has been most extensively studied, demonstrating 100% navigational success and reduced procedure time in 2 separate studies encompassing 35 patients, but faced challenges with therapeutic delivery in some instances [35, 36]. In a prospective multicenter study of 25 patients, Killer-Oberpfalzer et al. found device-related deficiencies occurred in 21% of aneurysm cases where coils, intrasaccular devices, or flow diverters were attempted [36]. This was thought to be caused by Bendit21’s larger inner diameter of 0.021” tram-tracking and compacting soft 0.014” coils during passage, leading to replacement with conventional microcatheters in some cases [36]. The LEONIS Mova steerable microcatheter, via manual tip angle adjustment up to 180°, has successfully treated cavernous sinus dural arteriovenous fistulas through occluded inferior petrosal sinuses, provided distal access for giant cavernous carotid artery aneurysm treatment with Pipeline Embolization Devices, and enabled direct coil placement in complex neurovasculature, though all applications required careful technique and had limitations including reduced tip visibility [37–39]. In contrast to these steerable microcatheter limitations, the DW24’s 0.024-inch diameter provided superior catheter support without delivery compromises and, based on our series, allowed its use across a wide range of intermediate catheters and microcatheters including Sim-2 Glide catheters, XT-27 microcatheters, Zoom intermediate catheters, and large-bore systems such as the 6 F Sofia aspiration catheter.

This study has several important limitations that must be considered when interpreting our findings. First, this represents a predominantly single-operator, single-center experience, with 91% (53/58) of cases performed by one operator, significantly limiting generalizability. Individual technique, skill level, and device preferences may have influenced both procedural outcomes and subjective usability assessments, particularly given that the DW24 system underwent a design iteration mid-study affecting 79% of cases. The absence of a control group using conventional guidewires prevents quantitative assessment of the steerable technology’s incremental value—while we report 100% technical success, we lack comparative data on procedure times, radiation exposure, or complication rates that would have been achieved with standard guidewires in matched cases. Our comparison between the DW24 and Artiria systems is inherently limited by multiple factors: vastly different sample sizes (48 vs. 10 cases), different wire diameters (0.024” vs 0.014”) conferring distinct mechanical properties, and lack of randomization to device selection. The smaller Artiria cohort provides insufficient power for meaningful statistical comparisons, and our subjective assessments of “poor shape retention” and “unreliable performance” were not quantified using objective measurements of torque transmission, tip deflection force, or shape memory. This comparative assessment reflects our institutional/operator experience with the available steerable wire options rather than a controlled head-to-head evaluation, and we anticipate that future development of multiple caliber steerable wires will allow for more appropriate comparisons within similar size categories.

Second, our study lacks the longitudinal follow-up necessary to assess long-term clinical outcomes or delayed complications, particularly relevant for complex cases like giant basilar aneurysms where durable patency remains critical. While we analyzed consecutive cases, independent multicenter validation would strengthen these findings and confirm generalizability across different operators and practice settings. Economic considerations were not evaluated despite their relevance for technology adoption decisions; the cost-effectiveness of steerable guidewires, particularly in complex cases where they may prevent procedural failure or reduce complications, requires formal analysis. Future prospective, randomized studies with standardized training protocols, objective performance metrics, and comprehensive economic analysis are needed to definitively establish the optimal role of steerable guidewire technology in neurointerventional practice.

## Conclusion

This study provides the largest single-center clinical experience with steerable guidewires in neurointerventional procedures and establishes first clinical evidence for the Artiria SmartGUIDE system. Our scoping literature review of steerable guidewires in neurointerventional procedures revealed only three previous studies with 65 total procedures, highlighting the limited reported evidence base for these devices. In our series, both systems achieved perfect technical success with excellent safety profiles; our preliminary observations suggest the DW24 may offer handling advantages for certain applications, though this requires validation in larger comparative studies. Our experience demonstrates that steerable guidewire technology enables successful navigation through challenging cerebrovascular anatomy where conventional approaches may fail, particularly in complex posterior circulation cases involving acute angulations and heavy catheter systems, while eliminating the need for repeated wire exchanges and maintaining established vascular access. These findings support the continued clinical evaluation and selective use of steerable guidewire technology in neurointerventional procedures.

## Supplementary Information

Below is the link to the electronic supplementary material.

Supplementary Material 1

## Data Availability

Patient-level data cannot be shared due to confidentiality restrictions and HIPAA regulations.

## Abbreviations

DW24: Drivewire 24
MMA: Middle Meningeal Artery
CTO: Chronic Total Occlusion
SCA: Superior Cerebellar Artery
PCA: Posterior Cerebral Artery
PICA: Posterior Inferior Cerebellar Artery
WEB: Woven EndoBridge
FDA: Food and Drug Administration
CE: Conformité Européenne
