# Supplementary material for: Improving catheter navigation in neurointerventional procedures: single-center insights on next-generation steerable guidewires
Source: Neuroradiology. 2026 Jan 24;68(5):1189–99. doi: 10.1007/s00234-026-03907-y (PMC13216097; doi:10.1007/s00234-026-03907-y)
Supplement: Supplementary file 1 — Supplementary Material 1 [file 234_2026_3907_MOESM1_ESM.docx]

| **Device Characteristic** | **Columbus Guidewire** | **Drivewire 24 (DW24)** | **Artiria SmartGUIDE** |
| --- | --- | --- | --- |
| **Physical Specifications** |  |  |  |
| Diameter | 0.014 inch | 0.024 inch | 0.014 inch |
| Total Length | 1925 mm (standard), 2000 mm (SR version) | 204 cm | 200 cm |
| Deflectable Tip Length | 15 mm | 15.7 mm | Not specified |
| Tip Bend Diameter | 8 mm (standard), 4 mm (SR version) | 5 mm when shaped into loop | Real-time adjustable |
| Radiopaque Length | Not specified | 40 cm | Not specified |
| Fluorosafe Marker Distance | Not specified | 100 cm from distal end | Not specified |
| **Control Mechanism** |  |  |  |
| Handle System | Pull/push handle; Automatic position lock | Proximally controlled deflectable tip; Automatic position maintenance | Manipulator handle system; Real-time deflection control |
| Tip Deflection Range | 4-8 mm radius depending on version | Variable radius control | Dynamic, real-time adjustable |
| Tip Control | Pull decreases radius (curves tip); Push increases radius (straightens tip) | Pull/push mechanism for tip shaping | Real-time deflection using manipulator |
| **Design Features** |  |  |  |
| Tip Characteristics | Very soft and malleable | Softer tip (similar to Synchro Support) | Soft atraumatic tip |
| Core Wire | Micro core wire connected to handle and tip | Internal support mechanism | Patented micromechanical system |
| Shaft Design | Stiff stainless-steel shaft with soft tip transition | Variable support profile | Not specified |
| Hydrophilic Coating | Standard | Hydrophilic distal tip | Not specified |
| **Catheter Compatibility** |  |  |  |
| Microcatheter Compatibility | 0.014" compatible microcatheters | 0.024" compatible microcatheters | 0.014" compatible microcatheters |
| Support Profile | Strong support when tip is shaped | Variable support across catheter range | Enhanced support when deflected |
| Wire Forerun | Can advance without wire forerun | Delivery without wire forerun | Eliminates need for wire forerun |
| **Regulatory Status** |  |  |  |
| CE Mark | ✓ | ✓ (Sept 2025) | Under evaluation |
| FDA Clearance | ✓ (as "Drivewire") | ✓ (2024) | ✓ 510(k) (May 2023) |
| **Manufacturer** |  |  |  |
| Company | Rapid Medical Ltd | Rapid Medical Ltd | Artiria Medical |
| Location | Yokneam, Israel | Yokneam, Israel | Borex, Switzerland |
| **Key Innovations** |  |  |  |
| Primary Innovation | First steerable guidewire with in-situ tip deflection | Larger diameter for improved support and torque transmission | Dynamic tip control with micromechanical system |
| Unique Features | Remote-controlled deflectable tip | Enhanced stability and torque vs 0.014" predecessor | Claims 3x faster navigation in cerebral arteries |
| **Reported Limitations** |  |  |  |
| Known Issues | Poor torquability, Handle detachment reported, Fragile construction | Suboptimal rotational response, Handle ergonomics | No published clinical limitations data |
| **Development Status** |  |  |  |
| Market Status | Production stopped | Currently available | First-in-human trials completed Q3 2023 |
| Clinical Data | Published clinical series available | Published clinical series available | No peer-reviewed clinical data published |

**Table S1.** Technical specifications and regulatory status of steerable guidewires in neurointerventional procedures.

| **Study Characteristic** | **Grin et al. 2025 (DW24)** | **von Hessling et al. 2022 (Columbus)** | **von Hessling et al. 2022 (Columbus)** | |
| --- | --- | --- | --- | --- |
|  |  |  |  |  |
| **Study Design** | Retrospective case series | Retrospective case series | Technical video/case demonstration |  |
| **Study Period** | October 2024 - April 2025 | August 2019 - December 2020 | 2021-2022 |  |
| **Institution** | NYU Grossman School of Medicine | Luzerner Kantonsspital, Switzerland | Luzerner Kantonsspital, Switzerland |  |
| **Sample Size** | 27 procedures in 26 patients | 36 patients | 2 illustrative cases |  |
| **Patient Demographics** |  |  |  |  |
| - Median Age | 59.8 years (19.0-83.9) | Not specified | Case-specific |  |
| - Gender Distribution | 69.2% female | 55.6% female (20F, 16M) | Mixed |  |
| - Access Route | Radial (48%), Femoral (44%), Both (8%) | Femoral arterial access (all cases) | Femoral access |  |
| **Indications** |  |  |  |  |
| - Aneurysm Treatment | 59.3% (16 cases) | 47.2% (17 cases) | 1 case (giant basilar tip aneurysm) |  |
| - Stroke Thrombectomy | 18.5% (5 cases) | 33.3% (12 cases) | - |  |
| - AVM/dAVF | 22.2% (6 cases) | 19.4% (7 cases) | 1 case (recurrent AComA aneurysm) |  |
| - Diagnostic Procedures | 7.4% (2 cases) | - | - |  |
| - Other procedures | - | Various (embolization, stenting) | - |  |
| **Technical Success Rate** | 92.6% (25/27 cases) | 97.2% (35/36 cases) | 100% (2/2 cases) |  |
| **Device-Related Complications** | None reported | Handle detachment (2 cases) | None reported |  |
| **Wire-Related Failures** |  |  |  |  |
| - Insufficient Support | 1 case (AVM embolization) | 1 case (kinked wire) | None |  |
| - Poor Torquability | 1 case (aneurysm access) | Major limitation noted | Noted as limitation |  |
| - Wire Damage | None reported | 5 cases (operator-related) | None |  |
| **Target Vessels Reached** |  |  |  |  |
| - MCA segments | Multiple cases | Multiple cases | 1 case |  |
| - Anterior cerebral artery | Multiple cases | Multiple cases | 1 case |  |
| - Posterior cerebral artery | Multiple cases | Multiple cases | 1 case (arising from aneurysm) |  |
| - ICA segments | Multiple cases | Multiple cases | - |  |
| - Venous sinuses | 2 cases | 1 case | - |  |
| - Basilar artery | - | Multiple cases | 1 case |  |
| **Specific Applications** |  |  |  |  |
| - Primary microwire | 51.9% (14 cases) | 97.2% (35 cases) | 1 case |  |
| - Secondary wire | 7.4% (2 cases) | 5.6% (2 cases) | 1 case |  |
| - Combination with other wires | 29.6% (8 cases) | 41.7% (15 cases) | 1 case |  |
| - PED post-processing | 4 cases | Flow diverter cases | - |  |
| **Catheter Compatibility** |  |  |  |  |
| - Microcatheters | VIA 27/33, Socrates 038, Midway 43 | SL-10 straight, Prowler select plus | SL-10 straight |  |
| - Intermediate Catheters | SOFIA 5, Phenom 27/PLUS, Navien 058 | Rebar 18/27, Phenom 27, Velocity | - |  |
| - Aspiration Catheters | RED 62, SOFIA PLUS | Various | - |  |
| **Procedural Outcomes** |  |  |  |  |
| - TICI 2B-3 (stroke cases) | 100% (5/5 cases) | High success rates reported | N/A |  |
| - Aneurysm flow stasis | Achieved in all aneurysm cases | Achieved in most cases | Achieved |  |
| - Device deployment success | High across all device types | High across all device types | Successful stent-assisted coiling |  |
| **Reported Advantages** |  |  |  |  |
| - Real-time tip deflection | ✓ | ✓ | ✓ (key feature demonstrated) |  |
| - Improved torque vs predecessor | ✓ (vs 0.014" Columbus) | Limited | Limited |  |
| - Variable support | ✓ | ✓ | ✓ |  |
| - No wire forerun required | ✓ | ✓ | ✓ |  |
| - Radiopaque tip visibility | ✓ | ✓ | ✓ |  |
| - Avoids aneurysm wall contact | ✓ | ✓ | ✓ (specifically demonstrated) |  |
| **Reported Limitations** |  |  |  |  |
| - Suboptimal rotational response | ✓ | ✓✓ (major limitation) | ✓ (noted) |  |
| - Handle ergonomics | ✓ | ✓ | ✓ |  |
| - Learning curve required | ✓ (short) | ✓ | ✓ |  |
| - Fragility concerns | None reported | ✓ (handle detachment) | None noted |  |
| - Tip response issues | ✓ (tightly curved shape) | ✓ | ✓ |  |
| **Specific Technical Innovations** |  |  |  |  |
| - Larger diameter benefits | Enhanced support and stability | N/A | N/A |  |
| - Improved torque transmission | vs 0.014" predecessor | Poor compared to standard wires | Poor compared to standard wires |  |
| - Softer tip design | Similar to Synchro Support | Very soft and malleable | Very soft |  |
| **Follow-up** |  |  |  |  |
| - Duration | Median 35 days (1-100) | Not systematically reported | Case-specific |  |
| - Clinical outcomes | 2 deaths (unrelated to device/pathology) | 1 death (complications unrelated to wire) | Good outcomes |  |
| **Operator Feedback** |  |  |  |  |
| - Learning curve | Short | Requires significant adaptation | Manageable with practice |  |
| - Overall assessment | Positive with noted limitations | Mixed (advantages vs major limitations) | Promising but limited by torquability |  |
| - Recommendation for use | Complex anatomy, specific indications | Conditional, specific scenarios only | Selected cases where tip control critical |  |

**Table S2.** Clinical studies literature review of steerable guidewires in neurointerventional procedures.
